# Supplementary material for: Developmental delay in attaining adult levels of motor excitability in children and adolescents with Tourette syndrome: a mega-analysis study
Source: bioRxiv. 2026 May 18:2026.05.14.724875. Preprint. [Version 2] doi: 10.64898/2026.05.14.724875 (PMC13228280; doi:10.64898/2026.05.14.724875)
Supplement: Supplement 1 [file NIHPP2026.05.14.724875v2-supplement-1.pdf]

bioRxiv preprint doi: https://doi.org/10.64898/2026.05.14.724875; this version posted May 18, 2026. The copyright holder for this preprint (which was not certified by peer review) is the author/funder, who has granted bioRxiv a license to display the preprint in perpetuity. It is made available under a CC-BY 4.0 International license.

Supplementary material:

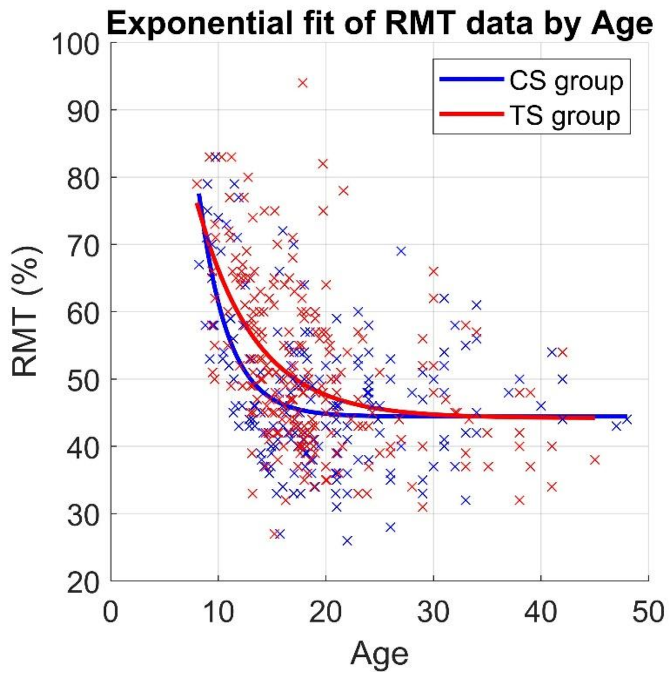

Figure S1: *Scatter plot showing individual RMT values as a function of age for the CS group (blue) and the TS group (red) when maximum age is restricted to 50 years. The data from each group was fitted using an exponential function. The solid blue line indicates the fitted function for the CS group and the red line for the TS group. Note both groups show a decrease in RMT with age during adolescence before reaching asymptote as adults.*

Effects of including a small number of older adults

To ensure that the data fitting process was not influenced by including a small or unequal number of older adults, we re-ran the analysis including only participants aged under 50 years (see Figure S1). This analysis confirmed the analyses reported above. Specifically, the analyses confirmed that the observed between-group difference in the rate of decrease in RMT with age during adolescence (k) was statistically significant (observed difference = 0.19, p = 0.005) and that the observed difference in the age at which each group attained adult values of RMT (asymptote) also remained statistically significant (CS asymptote reached at 12.4 years, TS asymptote reached at 24.8 years; p = 0.01). Once again, an independent groups t-test confirmed that once RMT values had plateaued at adult (asymptote) levels RMT they did not differ between the groups (Means: CS group 45.2 ± 8.5 years, TS group 44.5 ± 8.6 years; d = 0.07, t(190) = 0.7).

Effects of including a biphasic study without control group

One of the studies contributing to the pooled data was conducted using a TMS system that uses biphasic rather than monophasic pulses. TMS pulse type is known to significantly impact RMT [32], and given that this study also only contributed TS data we wanted to ensure our findings remain true with data from this site excluded (N= 48 TS participants). The analysis above was re-run using only data from the ten sites using monophasic TMS

bioRxiv preprint doi: https://doi.org/10.64898/2026.05.14.724875; this version posted May 18, 2026. The copyright holder for this preprint (which was not certified by peer review) is the author/funder, who has granted bioRxiv a license to display the preprint in perpetuity. It is made available under a CC-BY 4.0 International license.

pulses. It should be noted that this analysis is included for completeness. Biphasic pulses are known to reduce RMT which would converge from the pattern of results we see whereby individuals with TS have slightly higher RMTs on average with a decreased rate of reduction with age in the TS group.

<u>Excluding biphasic study without control group – Curve fitting</u>

An identical curve-fitting analyses was conducted with the biphasic study excluded (see Figure S1). The analyses confirmed that the observed between-group difference in the rate of decrease in RMT with age during adolescence (k) parameter was statistically significant (observed difference = 0.13, p = 0.05) and that the observed difference in the age at which each group attained adult values of RMT (asymptote) also remained statistically significant (CS asymptote reached at 12.4 years, TS asymptote reached at 18.7 years; p = 0.05). An independent groups t-test confirmed that once RMT values had plateaued at adult (asymptote) levels RMT did not differ between the groups (Means: CS group 45.2 ± 8.5 years, TS group 44.8 ± 8.2 years; d < -0.0001, t(208) = 0.995).

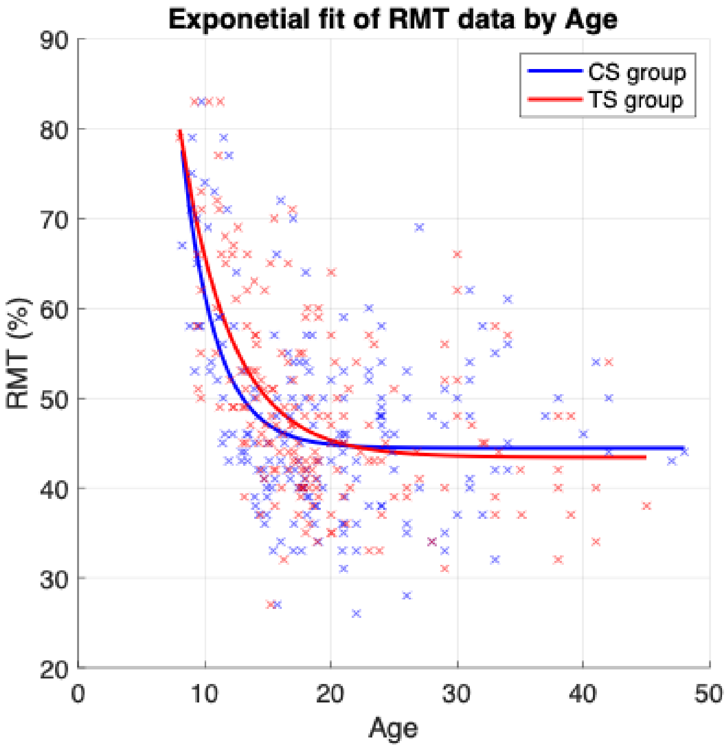

Figure S2: *Scatter plot showing individual RMT values as a function of age for the CS group (blue) and the TS group (red) for studies using monophasic TMS pulses only. The data from each group was fitted using an exponential function. The solid blue line indicates the fitted function for the CS group and the red line for the TS group. Note both groups show a decrease in RMT with age during adolescence before reaching an asymptote as adults.*
